# The Enhanced Formaldehyde-Sensing Properties of P3HT-ZnO Hybrid Thin Film OTFT Sensor and Further Insight into Its Stability

**DOI:** 10.3390/s150102086

**Published:** 2015-01-19

**Authors:** Huiling Tai, Xian Li, Yadong Jiang, Guangzhong Xie, Xiaosong Du

**Affiliations:** 1 State Key Laboratory of Electronic Thin Films and Integrated Devices, School of Optoelectronic Information, University of Electronic Science and Technology of China (UESTC), Chengdu 610054, China; E-Mails: jiangyd@uestc.edu.cn (Y.J.); gzxie@uestc.edu.cn (G.X.); xsdu@uestc.edu.cn (X.D.); 2 Tsinghua National Laboratory for Information Science and Technology (TNList), Institute of Microelectronics, Tsinghua University, Beijing 100084, China; E-Mail: lixian0317@mail.tsinghua.edu.cn

**Keywords:** TFT, P3HT-ZnO, formaldehyde gas sensor, heterojunction, stability

## Abstract

A thin-film transistor (TFT) having an organic–inorganic hybrid thin film combines the advantage of TFT sensors and the enhanced sensing performance of hybrid materials. In this work, poly(3-hexylthiophene) (P3HT)-zinc oxide (ZnO) nanoparticles' hybrid thin film was fabricated by a spraying process as the active layer of TFT for the employment of a room temperature operated formaldehyde (HCHO) gas sensor. The effects of ZnO nanoparticles on morphological and compositional features, electronic and HCHO-sensing properties of P3HT-ZnO thin film were systematically investigated. The results showed that P3HT-ZnO hybrid thin film sensor exhibited considerable improvement of sensing response (more than two times) and reversibility compared to the pristine P3HT film sensor. An accumulation p-n heterojunction mechanism model was developed to understand the mechanism of enhanced sensing properties by incorporation of ZnO nanoparticles. X-ray photoelectron spectroscope (XPS) and atomic force microscopy (AFM) characterizations were used to investigate the stability of the sensor in-depth, which reveals the performance deterioration was due to the changes of element composition and the chemical state of hybrid thin film surface induced by light and oxygen. Our study demonstrated that P3HT-ZnO hybrid thin film TFT sensor is beneficial in the advancement of novel room temperature HCHO sensing technology.

## 1. Introduction

Formaldehyde (HCHO) is a colorless volatile organic compound (VOC) with a strong, pungent-smelling that can be toxic, allergenic and carcinogenic [1]. Breathing air containing low levels of HCHO can cause damage to the human body, such as central nervous system damage, blood and immune system disorders, as well as bronchospasm, pneumonia and respiratory disease [2,3]. However, as an important industrial chemical, HCHO has been widely used in many industrial processes, such as in the production of fertilizer and paper, cosmetics and rubber industry [4,5]. Meanwhile, indoor HCHO is found in many products used every day around the house. Considering its widespread use, toxicity and volatility, exposure to HCHO is a significant consideration for human health. Therefore, developing portable, cheaper and resumable HCHO gas sensors operated at room temperature is in urgent need in both industrial and residential environments. Considerable efforts have been devoted to the development of different types of HCHO gas sensors, including resistive sensors composed of metal oxide semiconductor [6] and carbon nanotubes [7,8], quartz crystal microbalance (QCM) sensors [9], organic thin-film transistor (OTFT) sensors [10] and fiber-optic biochemical sensors [11]. Among various sensors, OTFT has been shown to be an inexpensive, portable and disposable diagnostic device because of its low cost, ease of fabrication and solution processibility, which are favorable for gas sensor application [12,13].

In an OTFT-based gas sensor, the active organic semiconductor layer in the channel is the critical element for the sensing performance [14]. The response behavior of OTFT sensor is strongly dependent on the active thin film properties such as morphology, doping level, surface trap density and grain boundaries, and so on. So far, most reports on OTFT-based gas sensors were based on the single sensitive materials such as small organic molecules [15,16] or conducting polymer [17,18]. Several approaches were demonstrated to improve the sensing performance of OTFT sensors by changing the grain structure of organic layer [19] or functional groups [20], or preparing bilayer sensing thin film [21]. Additionally, the concept of organic–inorganic hybrid material has been explored to obtain gas sensors with improved properties by taking full advantages of both component materials [22,23]. However, little work has been focused on employing of such hybrid materials as active layers in OTFT sensors, which could potentially combine the advantages of OTFT sensor with an enhanced sensing performance of hybrid materials.

Until now, various metal oxide including non-transition-metal oxides (SnO_2_, ZnO, *etc.*) and transition-metal oxides (NiO, WO_3_, *etc.*) have been used as HCHO-sensing materials. Among these materials, ZnO has attracted particular attention in gas sensor development for a long time due to its advantages of high sensitivity, simplicity in fabrication and low cost [6]. The ZnO-based gas sensors have been widely used in HCHO detection [24–26]. Recently, some initial results on organic–inorganic hybrid or bilayer thin films-based OTFT sensors were reported by our group using a spraying process [10,27,28]. A poly (3-hexylthiophene) (P3HT)-ferric oxide (Fe_2_O_3_) hybrid thin film HCHO OTFT sensor was demonstrated in [27]. A comparison analysis on P3HT-zinc oxide (ZnO) (P3HT-ZnO) hybrid and P3HT/ZnO bilayer films OTFT sensors was given in [28], which revealed that P3HT-ZnO hybrid film sensor exhibited optimum sensing properties. Meanwhile, an optimization on the process parameters such as P3HT: ZnO weight ratios and airbrushed masses for P3HT-ZnO film preparation were reported in [10].

This work is aimed to acquire a deep and comprehensive understanding of P3HT-ZnO hybrid thin film OTFT HCHO sensor. A thorough comparative analysis was performed between single P3HT and P3HT-ZnO hybrid thin film OTFT sensors in terms of morphological, compositional, electrical and gas-sensing properties. The HCHO-sensing mechanism of the hybrid thin film sensor was also investigated. Furthermore, the stability of P3HT-ZnO film sensor in the environment was measured, and X-ray photoelectron spectroscope (XPS) and atomic force microscopy (AFM) techniques were employed to understand the deterioration of sensor responses.

## 2. Experimental Section

### 2.1. Preparation of P3HT-ZnO Hybrid Thin Film OTFT Sensors

**Figure 1.**
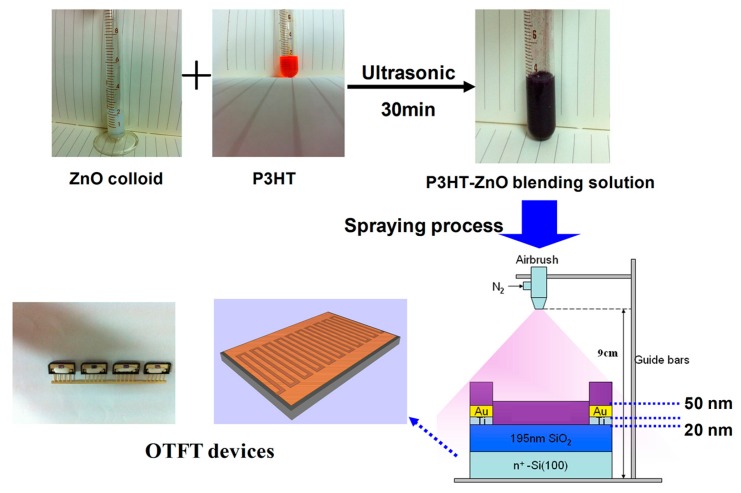
A schematic drawing of spraying process and organic thin-film transistor (OTFT) devices.

Details of experimental processes on OTFT sensor based on P3HT-ZnO hybrid thin film was reported in our earlier work in [10]. A schematic drawing of spraying process and OTFT devices is given in Figure 1. In short, P3HT (purchased from Luminescence Technology Corp.) chloroform solution was mixed with diluted ZnO nanoparticles (purchased from Sigma-Aldrich, 40 wt% dispersed in ethanol, <130 nm) to form the blending solution under ultrasonication, and then P3HT-ZnO hybrid thin film was spray-deposited on the bottom-contact OTFT device as the active layer. According to parameter optimization results in our previous work [10], optimal sensing-properties with maximum changes of threshold voltage and response value under 100 ppm HCHO exposure could be obtained when the weight ratio of P3HT:ZnO was 1:1 and the airbrushed mass of hybrid solution was 1 mL. These processing parameters were thus adopted for preparing P3HT-ZnO hybrid thin film in the present work. The deposited OTFT device was followed by heat treatment at 60 °C for 12 h in a vacuum until the solvent was completely evaporated. The pure P3HT film coated OTFT device was also prepared under the same condition for comparison. The channel width and length of both OTFT devices was 4000 and 25 μm, respectively.

### 2.2. Measurement Procedure and Characterization

**Figure 2.**
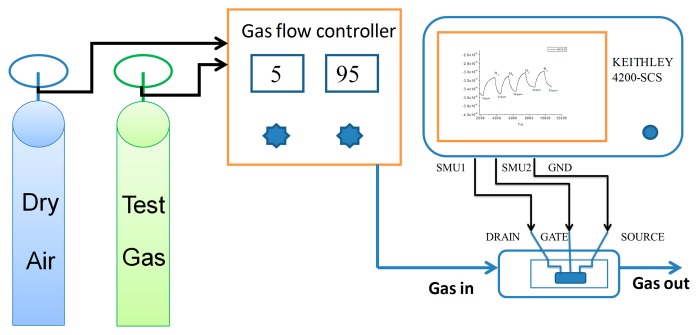
Schematic illustration of the experimental setup.

The measurements were carried out in a home-built testing system as shown in Figure 2. An image of the whole sealed testing chamber was inserted. The OTFT sensor was loaded inside the chamber under dry air atmosphere flow before testing to eliminate the ambient gases. Mass flow controllers (MFC) were employed to dilute and introduce HCHO vapors with a series of concentrations. All the measurement results were obtained in a bright air environment at room temperature. The source electrode of OTFT sensor was set to be common and the drain voltage (V_DS_) is swept from 0 to −60 V at gate bias (V_GS_) between 0 and −50 V in a step of −10 V. The saturation drain-source current (I_DS_) in the accumulation mode was monitored as a good gas-sensing property parameter when exposed to HCHO through Keithley 4200-SCS. The working point (V_DS_ = −50 V, V_GS_ = −30 V) of OTFT sensor was chosen in the saturation region of the output characteristics.

The morphology features of sensitive films were observed using a field emission scanning electron microscope (FESEM, FEI Inspect F, Hillsboro, OR, USA) and atomic force microscopy (AFM, Asylum Research MFP3D-Bio, Santa Barbara, CA, USA). Ultraviolet-Visible (UV-Vis) spectra were measured with UV-1700 pharmaspec (Shimadzu, Kyoto, Japan) in the range of 300∼1100 nm. The X-ray photoelectron spectroscope (XPS) analysis was performed with a commercial X-ray photoelectron spectrometer (Scienta ESCA-200, Uppsala, Sweden) using MgK_α_ X-ray source. The charging effect was corrected by using the binding energy of C1s signal at 285 eV.

## 3. Results and Discussion

### 3.1. Characterization of P3HT, ZnO and P3HT-ZnO Films

**Figure 3.**
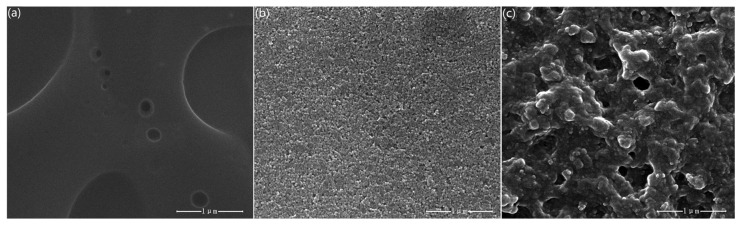
SEM graphs of sprayed (**a**) P3HT film; (**b**) ZnO film; (**c**) P3HT-ZnO hybrid film.

Figure 3 shows the typical SEM surface images of sprayed P3HT film, ZnO film and P3HT-ZnO hybrid film. It is seen that P3HT film exhibits discontinuous sheet structure with some cavities because of sprayed drops. ZnO nanoparticles appear spherical-like and the film surface is very compact. P3HT-ZnO hybrid thin film with a porous structure is contiguous and dense at the micro-scale and ZnO nanoparticles are dispersed in P3HT. However, the hybrid film also exhibits non-uniform morphology to some extent, which is assumed to be related to the imperfect blending uniformity and solubility of P3HT-ZnO mixture in solvents and sprayed drops, although the blending solution is good enough for the film process in the experiment. As compared to the single P3HT film, P3HT-ZnO hybrid film has larger surface area and thus provides more adsorption sites, which could contribute to a faster diffusion of gas molecules and a higher response value.

**Figure 4.**
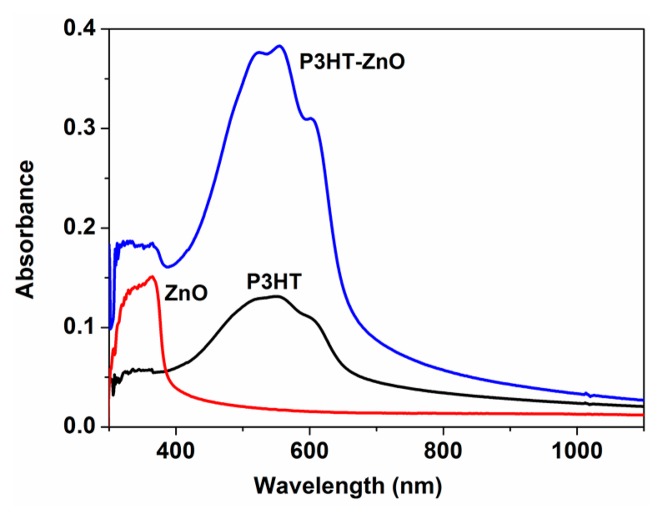
UV-Vis absorption spectra of P3HT, ZnO and P3HT-ZnO thin films.

**Figure 5.**
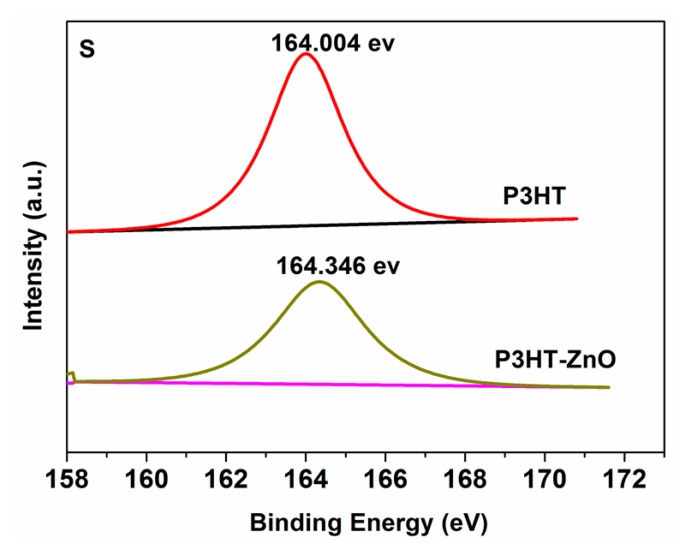
S 2p XPS spectra of P3HT and P3HT-ZnO thin films.

Figure 4 shows the UV-Vis absorption spectra of the P3HT film, ZnO film and P3HT-ZnO hybrid film. For the pure P3HT film, the spectrum has a peak at 550 nm and a shoulder at about 600 nm in the visible zone, which is attributed to π-π* transition [29]. ZnO nanoparticles thin film absorbs UV light in the wavelength range from 300–400 nm because of its wide-band-gap of about 3.37 eV [30,31]. The spectrum of P3HT-ZnO hybrid thin film exhibits the overlapping absorption bands of P3HT and ZnO, and the maximum adsorption value of hybrid film is twice as much as that of pure P3HT film, indicating the increased thickness of hybrid film according to the Beer-Lambert Law. No obvious blue or red shift is observed for the hybrid film from as shown in Figure 4, indicating that no distinct chemical interaction between P3HT and ZnO occurred. This is further supported by the S 2p XPS spectra of single P3HT and P3HT-ZnO hybrid films as shown in Figure 5. The peak of 164.004 eV corresponds to S 2p_3/2_ of the thiophene ring of P3HT [22], and no new peaks are found in the hybrid film. However, the S 2 p_3/2_ binding energy of hybrid film increases from 164.004 eV to 164.346 eV as compared to the pristine P3HT film, which is probably due to the synergetic effect between P3HT and ZnO components.

### 3.2. Electrical Properties of Prepared OTFT Sensors

**Figure 6.**
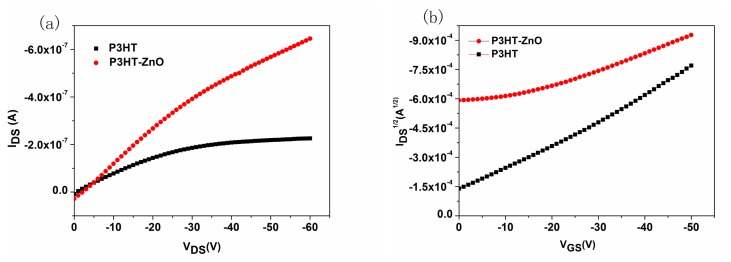
The typical (**a**) output (*V_GS_* = −30 V) and (**b**) transfer (*V_DS_* = −50 V) characteristics curves of P3HT and P3HT-ZnO films based OTFT sensors.

The typical output (*V*_GS_ = −30 V) and transfer characteristics (*V_DS_* = −50 V) curves of P3HT and P3HT-ZnO thin films based OTFT sensors are shown in Figure 6a,b, respectively. The two sensors exhibit a clear p-channel transistor behavior whereas the P3HT film OTFT possesses more obvious linear and saturation regions than the hybrid thin film device. The *I_DS_* in the saturated regime is given by the following equation:

(1) $$I_{DS}=\frac{W\mu C_{i}}{2L}{\left(V_{GS}-V_{th}\right)}^{2}$$

where C_i_ (17.7 nF/cm^2^ here) is the capacitance of dielectric layer [10], *W* = 4000 μm, *L* = 25 μm. The threshold voltage (V_th_) and field-effect mobility (μ) values could be extracted and calculated from Figure 6 and using Equation (1). A high *V_th_* value of about 40 V for P3HT-ZnO and −2 V for P3HT thin film OTFTs was graphically extrapolated from Figure 6b, respectively, and the mobility value of two devices was about 6.7 × 10^−5^ cm^2^/Vs and 1.6 × 10^−4^ cm^2^/Vs, respectively. Based on the SEM analysis, the worse TFT characteristics of P3HT-ZnO thin film device could be attributed to the low degree of morphological and structural order of hybrid film, and the discontinuous ZnO nanoparticles might also affect the carrier mobility. This is not ideal for TFT application which requires higher mobilities, however, the contiguous and porous morphology of the hybrid film is beneficial for gas sensing applications, as its large surface effective area with more absorption sites offers stronger interaction with HCHO gas molecules [32].

### 3.3. HCHO-Sensing Characteristics

**Figure 7.**
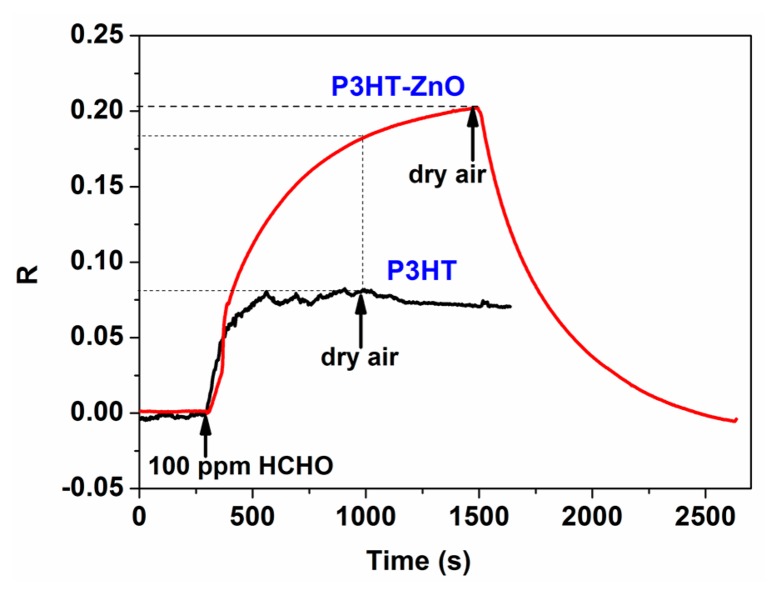
Real-time response curves of P3HT and P3HT-ZnO film OTFT sensors exposed to 100 ppm HCHO at room temperature.

**Figure 8.**
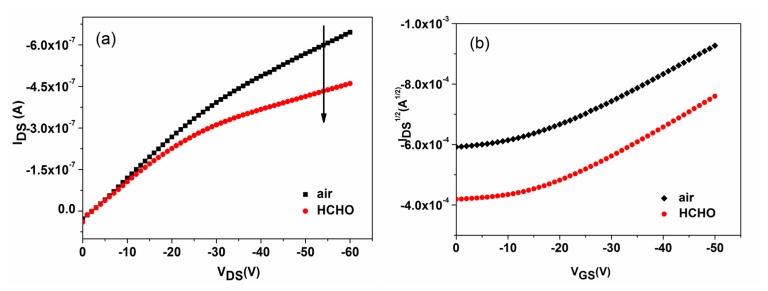
The (**a**) output and (**b**) transfer characteristics curves of P3HT-ZnO film OTFT sensor exposed to 100 ppm HCHO compared with those in air.

The response and recovery properties to analytes are the most direct and important characteristics for gas sensors. The real-time response curves of P3HT and P3HT-ZnO OTFT sensors exposed to 100 ppm HCHO at room temperature were measured and shown in Figure 7. The sensing response (*R*) is defined by *R* = (*I*_air_ − *I*_gas_)/*I*_air_, where *I*_air_ and *I*_gas_ is the I_DS_ in dry air and being exposed to tested gas, respectively. A positive value of R implies that the I_DS_ value decreases when the sensor is exposed to tested gas and *vice versa*. It is readily seen that an decrease of I_DS_ is induced in the presence of HCHO for the two sensors, and the response was changed by about 0.08 and 0.201 for P3HT and P3HT-ZnO thin film OTFT sensor, respectively, indicating a significant enhanced response by more than two times for the hybrid thin film sensor. Meanwhile, the P3HT-ZnO OTFT sensor could recover completely to the original baseline while the P3HT sensor exhibited very poor reversibility. Therefore, the P3HT thin film OTFT sensor was not investigated during our additional measurements. Figure 8 exhibited the output (*V_GS_* = −30 V) and transfer (*V_DS_* = −50 V) characteristics curves of the P3HT-ZnO hybrid thin film OTFT sensor exposed to 100 ppm HCHO compared with those in air. It can be observed clearly that the output I_DS_ decreases and the *V_th_* of the sensor shifts toward the negative direction under HCHO exposure, from which Δ*V_th_* of 23 V was extracted. Furthermore, the μ value increases from 6.7 × 10^−5^ cm^2^/Vs to 8.0 × 10^−5^ cm^2^/Vs, indicating the multi-parametric properties of OTFT gas sensors.

**Figure 9.**
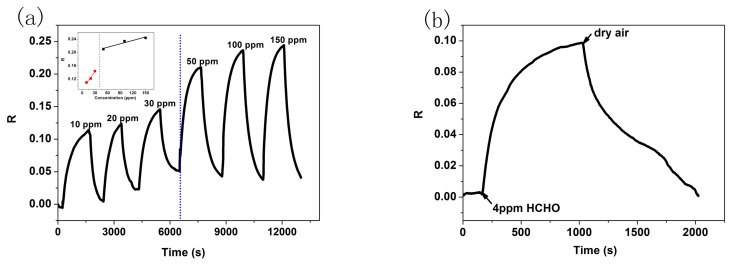
(**a**) The transient response of P3HT-ZnO hybrid film sensor when exposed to different HCHO concentrations, inset was the curve of response values *versus* concentration; (**b**) the detection limit (4 ppm) measurement curve at room temperature.

The transient response of P3HT-ZnO hybrid thin film OTFT sensor as a function of time is shown in Figure 9a when exposed to different exposure/evacuation cycles of HCHO at concentrations from 10 to 150 ppm, indicating a clear decrease of the current in all cases. The curve of sensing response values *versus* HCHO concentrations is given as inset in Figure 9a, in which the sectionalized linear fitting was performed for the low and high concentration range, respectively, indicating the good linearity degree of hybrid film sensor. Meanwhile, the detection limit of 4 ppm HCHO could be obtained for sensor as shown in Figure 9b. It was found that the sensor could not recover completely to their original baseline at the relatively low concentration, which is noted as baseline drift [16]. The causes for the baseline drift can be multiple. Firstly, there might be a discrepancy between the pre-exposure value and the recovered value during the initial exposures for gas sensors, and this effect is called conditioning as a result of residual gas molecules at low concentration, which is likely due to trapping of gas molecules within the hybrid thin film [33]. Secondly, there is likely baseline instability in ChemFETs including electrical, thermal and analyte-induced instabilities. For OTFTs, the electrical instability is usually the major cause of baseline drift, which is associated with charge trapping in organic films and insulator by direct tunnel model. Therefore, the phenomenon also might be partly ascribed to the electrical instability when exposed to HCHO at the beginning [34]. However, the clear understanding of underlying mechanism of these causes should be researched further.

The selectivity of OTFT sensors was investigated with some gases which might interfere with HCHO sensing, and the results showed a good specificity in our previously reported work [10]. However, a current decrease of sensor was observed when exposed to moisture (50% R.H.), and the response (0.2) was comparable to that for 50 ppm HCHO. The mechanism of this phenomenon was supposed to be that polar water molecules residing at the grain boundaries interact with hole carriers, or the diffusion of water molecules changes the intermolecular interactions in grain boundaries and increases the energy barrier for carrier transport [35]. Therefore, the interference of water on the prepared sensor was non-negligible for the practical application. Accordingly, three possible approaches are proposed to eliminate the effect of moisture. (1) A gas filtering system could be designed to remove moisture from the sensing chamber; (2) Humidity compensation algorithm might be implemented in the read-out signal processing system of OTFT sensors; (3) The encapsulation of OTFT devices might be processed; that is, a moisture absorption layer (such as typical porous organic fiber humidity-sensitive materials) could be installed inside the encapsulation shell with the cover. When the sensor is exposed to HCHO gas, the cover could be removed and the moisture would be absorbed by the moisture absorption layer. HCHO molecules could penetrate this layer and react with the sensitive layer of OTFT sensor. However, the employment of proper technique should be studied further. The performance comparisons of our OTFT sensor to various kinds of sensors recently reported are summarized in Table 1. It can be seen that the present OTFT sensor can deliver responses at room temperature, unlike the ZnO-based sensor that detects HCHO at higher working temperatures. Meanwhile, such OTFT sensors work as reversible multi-parameter devices by a simple and feasible spraying process with low cost.

### 3.4. The HCHO-Sensing Mechanism of Hybrid Thin Film OTFT Sensor

The response of P3HT thin film to HCHO gas molecules could be explained via multiple factors. In general, the channel current would be changed by charge doping or trapping due to analytes for OTFT sensors [14]. Since the channel length (25 μm here) is much larger than the grain size of P3HT layer, the gas sensing mechanism should be described as polar HCHO molecules adsorbed on the active layer or sites dipole-induced charge trapping at grain boundaries through noncovalent bonds (such as hydrogen bonds and π interactions), which leads to a decrease in the current [20,38]. From the point of view of charge transfer interactions, the decreased current could be attributed to donated or injected electrons from HCHO molecules to P-type P3HT layer, for HCHO is a reductive gas and acts as electron-donor, leading to the decreased hole carriers concentration in P3HT material [22].

**Figure 10.**
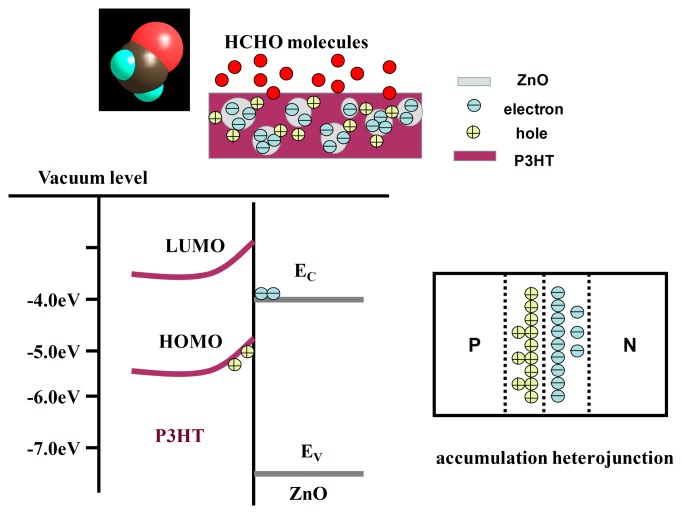
The schematic diagram showing the energy level and charge accumulation of P3HT-ZnO composite.

It is obvious that introducing ZnO nanoparticles into P3HT has led to the improved HCHO-sensing property. The sensing performance should be affected by three independent factors, *i.e.*, receptor function, transducer function and utility [39]. The response difference of P3HT and P3HT-ZnO thin film sensors is analyzed as follows. (i) It has been proposed that the response behavior is strongly dependent on film morphology. Based on SEM images and analysis, the surface of P3HT-ZnO hybrid thin film is richer in HCHO molecules binding groups than P3HT thin film, so that HCHO binding probability is higher. Meanwhile, gas molecules could access the grains located at inner sites for the porous structure, that is, HCHO molecules are likely to bind, and also diffuse into the bulk when intercalating inside defects of the hybrid layer. The porous morphology of P3HT-ZnO hybrid film also promotes desorption of gas molecules compared with sheet structure of P3HT film, partly resulting in the better reversibility of hybrid film sensor; (ii) The donor-acceptor like complex between ZnO nanoparticles and P3HT might be formed. The energy diagram of P3HT-ZnO composite is plotted in Figure 10, in which E_LUMO_ and E_HOMO_ is the energy level of the lowest unoccupied molecular orbital (LUMO, −3.2 eV) and the highest unoccupied molecular orbital (HOMO, −5.0 eV) of P3HT, respectively [31]. *E_V_* (−7.6 eV) and *E_C_* (−4.4 eV) is the energy level of the valence band and the conduction band of ZnO, respectively [40]. So, an accumulation p-n heterojunction structure is formed in the P3HT-ZnO composite [41], as shown in Figure 10; P3HT acts as the dominant charge transfer with HCHO gas molecules, and more holes will accumulate in the heterojunction region for the balance after HCHO gas molecules interact with P3HT, which should facilitate charge transfer and result in better gas-sensing properties. Another possible mechanism for enhanced HCHO response and reversibility of hybrid film could be associated with lower potential barrier of P3HT surface because of the band bending, leading to the reduction of activation energy and enthalpy of physisorption for HCHO gas molecules [42], and thus adsorbed HCHO molecules are more easily displaced by oxygen at adsorption sites when HCHO was removed by dry air.

### 3.5. The Stability Investigation of P3HT-ZnO Hybrid Thin Film Sensor

Polymer-based devices are usually prone to the deterioration of performance upon long-term exposure in the presence of natural environment [43]. Our prepared P3HT-ZnO hybrid thin film sensor was stored in an open chamber at room temperature for two weeks in order to investigate the influence of light and air on gas-sensing characteristics. The variation in the sensing response is studied and the possible reason is further analyzed by SEM, AFM and XPS characterizations in this section.

**Figure 11.**
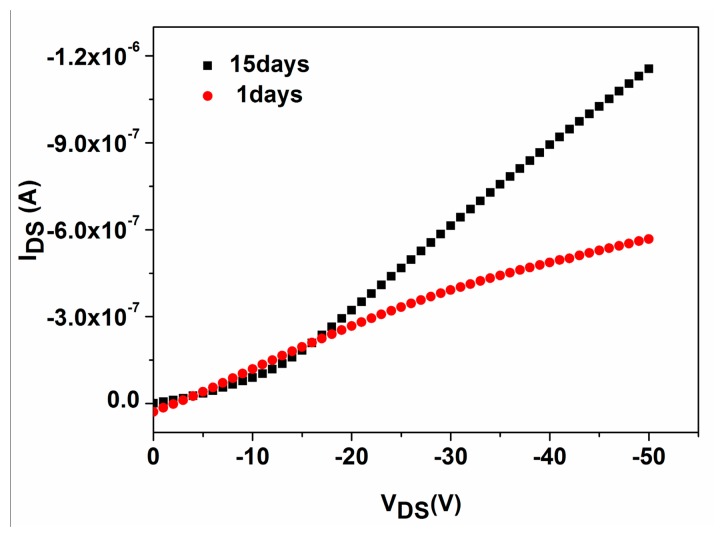
The typical output characteristic curves (*V_GS_* = −30V) of P3HT-ZnO hybrid thin film sensor stored after 1 and 15 days.

**Figure 12.**
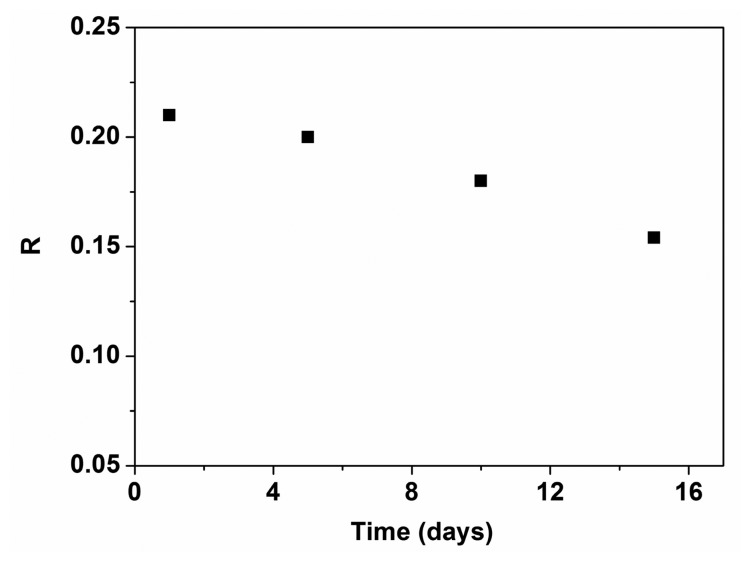
The response of P3HT-ZnO sensor for 1, 5, 10 and 15 days when exposed to 100 ppm HCHO.

Figure 11 shows the output characteristic curves (*V_GS_* = −30 V) of P3HT-ZnO hybrid thin film OTFT sensor stored after 1 and 15 days, revealing that the I_DS_ of the sensor after 15 days becomes higher than that of the sensor after 1 day. However, the p-channel transistor behavior of the sensor stored 15 days becomes weak. Figure 12 shows the response of the sensor on exposure to 100 ppm HCHO under ambient conditions for 1, 5, 10 and 15 days, showing that the response value decreased by approximately 25% after 15 days.

**Figure 13.**
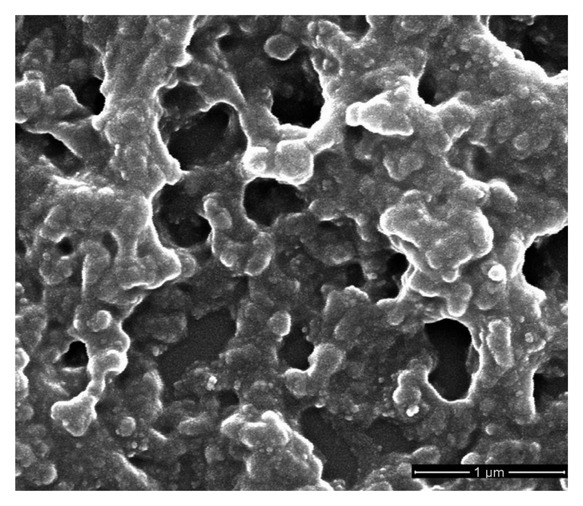
SEM graphs of P3HT-ZnO hybrid thin film stored after 15 days.

**Figure 14.**
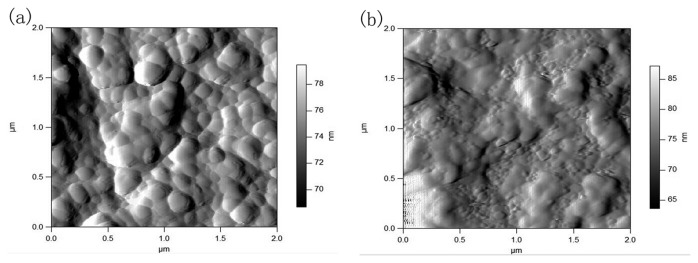
AFM amplitude images of P3HT-ZnO hybrid thin film stored after (**a**) 1 and (**b**) 15 days.

**Figure 15.**
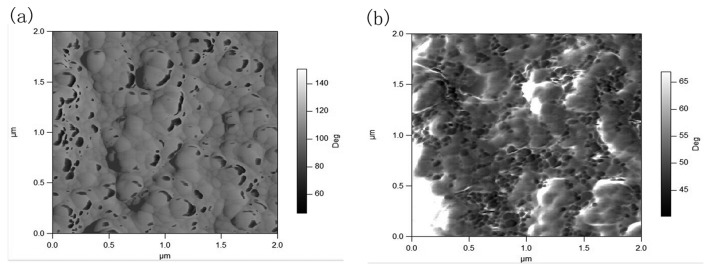
AFM phase images of P3HT-ZnO hybrid thin film stored after (**a**) 1 and (**b**) 15 days.

The sensing performance of OTFT sensors could be influenced by the film composition, topographic features, and grain boundaries of sensitive thin films and so on. Therefore, the microstructures and chemical states of P3HT-ZnO hybrid thin film are investigated by SEM, AFM and XPS in order to better understand the performance variation of sensor. Figure13 shows SEM image of P3HT-ZnO hybrid thin film stored after 15 days. Compared to the previous SEM image of P3HT-ZnO film as given in Figure 3c, a similar surface morphology with three-dimensional structures and many holes is observed and no obvious difference exists. Next, the surface topography of hybrid thin film stored after 1 and 15 days is further characterized with tapping mode AFM, and amplitude images and phase images of P3HT-ZnO hybrid thin film are shown in Figures 14 and 15, respectively. For the film stored after 1 day, the AFM image shows a relatively smooth and homogeneous grains surface with a RMS surface roughness of 41.769 nm. However, the P3HT-ZnO hybrid thin film shows the appearance of ZnO components on surface layer after 15 days as shown in Figure 15b, in which the darker spots should be ZnO nanoparticles, while the light background is the P3HT thin film, with a surface roughness of 72.092 nm.

**Figure 16.**
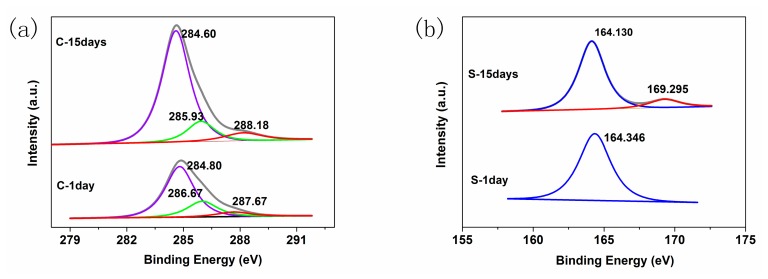

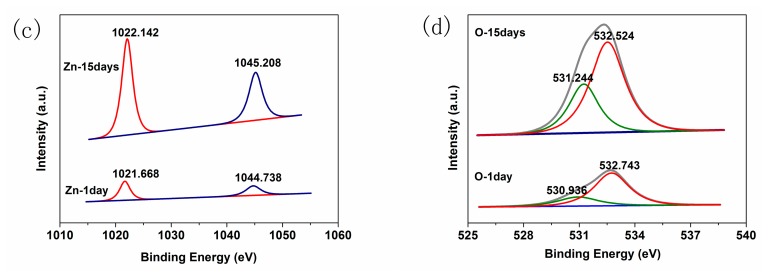
XPS spectra of (**a**) C; (**b**) S; (**c**) Zn and (**d**) O elements for P3HT-ZnO hybrid thin film stored after 1 and 15 days.

The element composition and chemical state of hybrid thin film surface could be examined by XPS. Therefore, the high-resolution of C, S, Zn and O XPS spectra of P3HT-ZnO hybrid thin film are measured for obtaining further insight into its stability, and the spectral survey results are recorded in Figure 16. For the pristine P3HT-ZnO hybrid thin film sample, gaussian-shaped three peaks are found at 284.8, 286.0 and 287.7 eV in C1s spectrum as shown in Figure 16a, which are assigned to C-C/C-H, C-S/C-O and C=O, respectively [44]. After 15 days, the subpeak at 287.7 eV of C1s spectrum exhibited a shift toward the higher binding energy direction, and a broadening on the high binding energy side is observed. For quantification, the area under each subpeak of C1s spectrum is determined and the oxidized carbon ratio is calculated. It is found that the ratio improves from 7.41%–8.12%, indicating that C=O group increases and the carbon atoms are further oxidized. The evolution of S 2p signal is given in Figure 16b. The S 2p spectrum exhibits a well-resolved doublet with a unique component at 164.346 eV (S 2p_3/2_), which is consistent with a single chemical state of sulphur in the polymer chain [45]. A new peak appears centered at 169.295 eV which is shifted by about 5.1 eV toward the higher binding energy relative to the original S 2 p_3/2_ signal after 15 days. This new signal should be assigned to be a sulphone (-SO_2_-) and is indicative for the oxidation of sulphur atoms of thiophenic ring [45,46]. Therefore, it is inferred from C1s and S 2p spectra that the degradation of aged P3HT-ZnO hybrid thin film occurred in certain degree resulting from the oxidation of P3HT due to the presence of the light or oxygen in the environment. In addition, Figure 16c exhibited the Zn 2p_3/2_ and Zn 2p_1/2_ subpeaks of ZnO located at 1021.7 eV and 1044.8 eV for the original hybrid thin film, respectively [47]. However, the Zn 2p signals shift to binding energies which are higher than those for pristine Zn by 0.5 eV after storage of 15 days, indicating the further oxidation of Zn element, which was also probably related to the morphology instability based on AFM results [48]. For O element, the subpeaks at 530.9 and 532.7 eV in O1s spectrum of Figure 16d are assigned to Zn-O and C-O, respectively, and the O 1s subpeak at 532.7 eV of P3HT shifts toward lower binding energy. It is assumed that the oxygen molecules bound to the π-system of P3HT trap an electron and these oxygen species probably act as quenching sites for excited states of P3HT, resulting in a degradation of P3HT component [43]. The element contents of P3HT-ZnO film *versus* ageing duration are summarized in Table 2. One can notice the significant decrease of C and S elements contents, whereas the contents of O and Zn elements increase in the sample, indicating the increase of Zn element on the surface layer of hybrid thin film and the degradation of P3HT component.

It has been considered that most mobile charge carries might be concentrated near the bottom layer at the dielectric–semiconductor interface for OTFT devices [20]. According to AFM and XPS analyses, ZnO nanoparticles content increases on the top surface of the hybrid layer, indicating that the influence of ZnO nanoparticles on the charge transport along the conducting channel gets weakened; meanwhile, it is supposed that oxygen molecules trap electrons which leave behind mobile holes. Therefore, the saturation source-drain current of P3HT-ZnO hybrid thin film OTFT increases. However, the degradation of P3HT and the increased content of ZnO nanoparticles on the surface affect the sensing characteristics of the OTFT sensor. For the application of sensing devices, the active layer surface should be rich in HCHO molecule binding groups [33], but the grain boundary effect of P3HT on the film surface is reduced for the degradation of P3HT component as seen from our AFM and XPS results. On the other hand, exposure to oxygen and light for a long-time induces the shift of HOMO level position to lower binding energies, accompanied by an increase of the work function [43]. As a result, it is assumed that the high current and worse sensing performance of the sensor stored after 15 days should be attributed to the component instability of hybrid thin film surface and the degradation of P3HT material when exposed to light and air. However, the response value of pure P3HT decreased by almost 35% when exposed to 100 ppm HCHO, indicating that the performance deterioration of hybrid film sensor is less than that of single P3HT film one, which should be attributed to the unchanged porous structure of P3HT-ZnO hybrid film based on SEM results. Anyway, the proper encapsulation of sensors should be developed for partly eliminating the effect of environmental factors on gas-sensing characteristics and the thin film process should also be further improved.

## 4. Conclusions

In summary, P3HT-ZnO nanoparticles' hybrid thin film was deposited on the OTFT device by a simple solution spraying route for preparing HCHO gas sensors at room temperature. The improved HCHO sensing properties with higher response value and better reversibility were obtained compared to pristine P3HT thin film sensor, which was attributed to three-dimensional porous morphology and accumulation p-n heterojunction structure of P3HT-ZnO hybrid thin film. However, the decreased response value of prepared sensor in the presence of natural environment was a matter of concern. The SEM, AFM and XPS analyses' results demonstrated that component instability of the film surface and the degradation of P3HT material occurred when exposed to light and air, although no obvious surface morphological change was observed. Further work will be focused on optimizing the preparation process of hybrid thin film and developing new inorganic–organic hybrid/composite gas-sensing thin films for OTFT sensors. This work may open up new opportunities for fabricating OTFT HCHO sensors operated at room temperature based on organic–inorganic hybrid thin film after further development.

## Figures and Tables

**Table 1.** Brief summary of results reported for various types of HCHO sensors.

**Reference**	**Sensor Types**	**Sensitive Materials**	**Preparation Method**	**Operating Temperature (°C)**	**Detection Range (ppm)**
[6]	resistive	ZnO-MnO_2_	screen-printing + solution growth	320	0–300
[24]	resistive	Mn-doped ZnO nanorods	PECVD	400	0–205
[25]	resistive	ZnO nanopowders	microwave heating	210	0.001–1000
[36]	resistive	ZIF-67	---	150	5–500
[7]	resistive	MWCNTs with amino-group	mounted suspension	RT	0.02–0.2
[8]	conductance	TFQ functionalized SWNT	self-assembly + dropping	RT	0.15–5
[9]	QCM	PEI modified chitosan membrane	Electrospinning	RT	5–185
[37]	QCM	PEI/PVA	Electrospinning	RT	10–255
This work	OTFT	P3HT-ZnO	spraying	RT	4–150

Note: ZIF: zeolitic imidazolate framework; TFQ: tetrafluorohydroquinone; PEI/PVA: polyethyleneimine/poly (vinyl alcohol); ---: not referred.

**Table 2.** The element contents of P3HT-ZnO film surface for 1 day and 15 days.

**Element Content (%)**	**C**	**O**	**S**	**Zn**
1 days	73.46%	15.05%	9.54%	1.96%
15 days	68.36%	22.13%	4.49%	5.02%
